# ClinEpiDB: an open-access clinical epidemiology database resource encouraging online exploration of complex studies

**DOI:** 10.12688/gatesopenres.13087.2

**Published:** 2020-04-06

**Authors:** Emmanuel Ruhamyankaka, Brian P. Brunk, Grant Dorsey, Omar S. Harb, Danica A. Helb, John Judkins, Jessica C. Kissinger, Brianna Lindsay, David S. Roos, Emmanuel James San, Christian J. Stoeckert, Jie Zheng, Sheena Shah Tomko

**Affiliations:** 1 Infectious Diseases Research Collaboration, Kampala, Uganda; 2 Department of Biology, University of Pennsylvania, Philadelphia, PA, 19104, USA; 3 Department of Medicine, San Francisco General Hospital, University of California, San Francisco, CA, 94110, USA; 4 Center for Tropical & Emerging Global Diseases, University of Georgia, Athens, GA, 30602, USA; 5 Department of Genetics, University of Georgia, Athens, GA, 30602, USA; 6 Institute of Bioinformatics, University of Georgia, Athens, GA, 30602, USA; 7 Kwazulu-Natal Research and Innovation Sequencing Platform, Durban, South Africa; 8 Department of Genetics, University of Pennsylvania School of Medicine, Philadelphia, PA, 19104, USA; 9 Institute for Biomedical Informatics, University of Pennsylvania, Philadelphia, PA, 19104, USA

**Keywords:** ClinEpiDB, Epidemiology database, FAIR data, Data visualization, Infectious diseases, Malaria, Enteric disease

## Abstract

The concept of open data has been gaining traction as a mechanism to increase data use, ensure that data are preserved over time, and accelerate discovery. While epidemiology data sets are increasingly deposited in databases and repositories, barriers to access still remain. ClinEpiDB was constructed as an open-access online resource for clinical and epidemiologic studies by leveraging the extensive web toolkit and infrastructure of the Eukaryotic Pathogen Database Resources (EuPathDB; a collection of databases covering 170+ eukaryotic pathogens, relevant related species, and select hosts) combined with a unified semantic web framework. Here we present an intuitive point-and-click website that allows users to visualize and subset data directly in the ClinEpiDB browser and immediately explore potential associations. Supporting study documentation aids contextualization, and data can be downloaded for advanced analyses. By facilitating access and interrogation of high-quality, large-scale data sets, ClinEpiDB aims to spur collaboration and discovery that improves global health.

## Introduction

Large-scale epidemiological data sets offer immense potential for secondary data discovery and translational research provided the data are Findable, Accessible, Interoperable, and Reusable (FAIR) ( Wilkinson *et al.,* 2016). Data repositories such as Dryad, dbGaP, and to a more limited extent ICPSR support the deposition of epidemiology data and metadata for download and secondary use by other researchers. A few recent studies such as Child Health and Mortality Prevention Surveillance (CHAMPS) have taken data sharing a step further and allow open access to aggregate data and online data visualization tools even as the study continues and the database is regularly updated with new data. The Clinical and Epidemiology Database (ClinEpiDB) resource was developed within this landscape as an open-access online tool to help investigators quickly and easily explore data from complex epidemiological studies and distinguishes itself from repositories and study-specific websites in two key ways: 1) ClinEpiDB maps data to common ontologies, creating a unified semantic framework that applies to all integrated studies, even those with different disease foci. 2) That framework underpins the website, where investigators are encouraged to explore data online through interactive tables, graphs, and an intuitive visual query interface, reducing the time and effort required to determine if specific data are available within one or multiple studies and worth further analysis. While some repositories have integrated tools like Survey Documentation and Analysis (SDA, Institute of Scientific Analysis) for online analysis, these tools may only be available for particular data sets and access to data may be restricted. A distinguishing feature of ClinEpiDB is that tools and visualizations are available for all studies, and aggregate data is generally publicly accessible.

For the initial prototype of ClinEpiDB, socioeconomic, demographic, clinical, and other data from the Program for Resistance, Immunology, Surveillance and Modeling of Malaria in Uganda (PRISM) ( Kamya *et al.,* 2015), an International Center of Excellence for Malaria Research (ICEMR) ( Rao, 2015), was loaded into a relational database, leveraging infrastructure from EuPathDB (now VEuPathDB, reflecting a merger with VectorBase ( Giraldo-Calderón *et al.,* 2015)), a collection of databases supporting multi-omics research on eukaryotic microbial pathogens, relevant non-pathogenic species, and selected hosts ( Aurrecoechea *et al.,* 2017). Private release of the prototype to PRISM data providers prompted web tool optimization for settings with limited internet connectivity and led to rapid appreciation of the potential to facilitate data exploration by the full investigation team and raise study awareness. As a result, the PRISM study was publicly released in February 2018, even as primary publications on the data were still in preparation. The PRISM study was followed by release of ten additional studies ( Table 1), including the Global Enteric Multicenter Study (GEMS) ( Kotloff *et al.,* 2013) and the Etiology, Risk Factors, and Interactions of Enteric Infections and Malnutrition and the Consequences for Child Health study (MAL-ED) ( Acosta *et al.,* 2014). Additional releases containing data on malaria, enteric, respiratory, and other major global health priorities are scheduled for 2019–2020 and beyond.

**Table 1.** Studies publicly available via ClinEpiDB as of October 2019.

Study abbreviation (reference)	Study design (time frame)	Research focus	Record types (# records)	Search types	Release date/ access level
PRISM ( Dorsey *et al.,* 2018; Kamya *et al.,* 2015)	Longitudinal cohort (2011–2017)	Incidence of acute malaria and parasite prevalence at three sites in Uganda with differing exposure to mosquito vectors	Household (331) Participant (1421) Observation (48,722) Entomology (17,081)	Household Participant Observation Entomology	Feb 2018/Public
GEMS ( Gates Enterics Project *et al.,* 2018; Kotloff *et al.,* 2013)	Case-control with 60-day follow-up (2007–2011)	Cause, incidence, and impact of moderate-to- severe diarrhea in children from Bangladesh, the Gambia, India, Kenya, Mali, Mozambique, and Pakistan	Household (43,573) Participant (22,567) Observation (60,958)	Participant	Dec 2018/Protected
GEMS1A ( Gates Enterics Project *et al.,* 2019b; Kotloff *et al.,* 2019)	Case-control with 60-day follow-up (2011–2013)	Cause, incidence, and impact of less severe diarrhea in children from Bangladesh, the Gambia, India, Kenya, Mali, Mozambique, and Pakistan	Household (22,770) Participant (14,242) Observation (36,009)	Participant	Mar 2019/ Protected
India ICEMR longitudinal ( Carlton *et al.,* 2019a; Das *et al.,* 2012)	Longitudinal cohort (2013–2015)	Prevalence and incidence of malaria at two sites in India with varied transmission settings	Household (110) Participant (397) Observation (1249)	Household Participant Observation	Mar 2019/Public
MAL-ED ( Acosta *et al.,* 2014; Spiro *et al.,* 2019)	Longitudinal cohort (2009–2014)	Etiology, risk factors and interactions of enteric infections and malnutrition in children from Bangladesh, Brazil, India, Nepal, Pakistan, Peru, South Africa and Tanzania	Household (12,233) Participant (2145) Observation (1,384,323)	Participant Observation	Mar 2019/Protected
GEMS1 HUAS/HUAS Lite ( Gates Enterics Project *et al.,* 2019a; Nasrin *et al.,* 2013)	Household survey (2007–2010)	Utilization of and attitudes towards healthcare services. Survey conducted in conjunction with GEMS1	Household (133,659) Participant (133,659) Observation (133,659)	Participant	Apr 2019/Protected
GEMS1A HUAS Lite ( Gates Enterics Project *et al.,* 2019c)	Household survey (2010–2011)	Utilization of and attitudes towards healthcare services. Survey conducted in conjunction with GEMS1A	Household (62,193) Participant (62,193) Observation (62,193)	Participant	Apr 2019/ Protected
India ICEMR cross-sectional ( Carlton *et al.,* 2019b; van Eijk *et al.,* 2016)	Cross-sectional survey (2012–2014)	Prevalence of malaria at three sites in India with varied transmission settings	Household (1393) Participant (3267) Observation (3442)	Household Participant Observation	Apr 2019/ Public
India ICEMR fever surveillance ( Carlton *et al.,* 2019c; Rao *et al.,* 2019)	Health center surveillance (2016–2017)	Etiology of acute febrile illness in patients without malaria	Participant (954) Observation (962)	Participant	Apr 2019/ Public
Amazonia ICEMR Peru ( Rosas-Aguirre *et al.,* 2017; VinetzAmazonia ICEMR Peru *et al.,* 2019)	Longitudinal cohort (2012–2015)	Prevalence and incidence of malaria in disparate transmission settings	Household (487) Participant (2445) Observation (2,050,603)	Household Participant Observation	Jul 2019/ Protected
South Asia ICEMR ( Chery *et al.,* 2016; Rathod *et al.,* 2019)	Health center surveillance (2012–2017)	Correlates of clinical malaria severity and parasite phenotypes and genotypes	Participant (1546) Observation (4995)	Participant	Jul 2019/ Protected

[i] PRISM, Program for Resistance, Immunology, Surveillance and Modeling of Malaria; GEMS, Global Enteric Multicenter Study; HUAS, Healthcare Utilization and Attitudes Survey; ICEMR, International Centers of Excellence for Malaria Research; MAL-ED, Etiology, Risk Factors, and Interactions of Enteric Infections and Malnutrition and the Consequences for Child Health.

The resulting ClinEpiDB resource facilitates easy access and exploration of epidemiologic study design details and data for each study that is loaded. Study methodology, supporting documentation, and attribution are accessible through study pages. The ClinEpiDB user interface enables point-and-click interrogation of diverse data types where variables are displayed as interactive tables and histograms, allowing users to contextualize and identify subsets of data and visualize and analyze the results. For example, users can explore the impact of geographic location, mosquito exposure, and housing design on the frequency of acute malaria versus asymptomatic *Plasmodium* infection in the PRISM study. Entire data sets or filtered subsets of data can be downloaded for more advanced analyses. For data sets that require advanced security, ClinEpiDB offers a tiered data access system. All ClinEpiDB data sets released to date allow complete access to aggregate data and visualization tools, but some studies require that data access requests must be approved in order to view and download disaggregate data.

## Methods

### Ethical statement

The ClinEpiDB platform has received approval from the University of Pennsylvania under IRB#7, Protocol #828806. All studies included in ClinEpiDB have undergone ethical approval at applicable institutions prior to data collection (ClinEpiDB is generally not involved in this process). Data providers also obtain approval from their institutions to have their data hosted on ClinEpiDB. Community engagement programs have not yet been undertaken to assess study participants’ attitudes towards data sharing via the ClinEpiDB platform.

### Implementation

ClinEpiDB integrates studies conducted by various primary research groups and can accommodate a variety of study designs including observational studies (surveillance, cross-sectional, longitudinal cohort, and case-control) and randomized control trials. Researchers supply flat data files along with data dictionaries, data collection forms, and protocols to help contextualize the data. Variables within the data set may contain categorical, continuous, discrete, or free text data.

**Figure 1.**
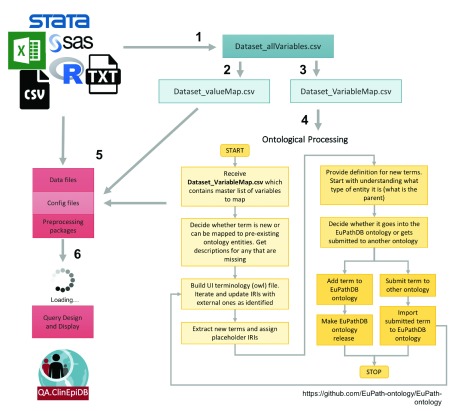
Pipeline for processing studies. (1) The ClinEpiDB team generates an “allVariables” file from the raw data files, data dictionaries, and data collection forms that contains all variables collected as part of the study and indicates whether each variable will be displayed on the website or not. This file is used to make (2) a “valueMap” file that maps coded categorical values to descriptive terms to be displayed on the website and (3) a “variableMap” file that maps variables to existing ontology terms and labels for display on the website. (4) The “variableMap” file is further processed by the ontology team and new ontology terms are created as needed. (5) All files are passed to the data loading team to pre-process the data, shift dates based on a random number algorithm, and create ISA files to load into the GUS4 database. (6) Once files are loaded, the data appear on an access-restricted website. Any additional searches required by a study are designed and implemented.

Once the data are received, a series of files are constructed according to a standard operating procedure to process the variables, map them to ontology terms, and map coded categorical values to the descriptive terms displayed on the website ( Figure 1). In rare cases where personally identifying variables – such as participant names and addresses – are included in the files received, those variables are removed before files are stored on a private file system only accessible to the data loading team to ensure participant confidentiality. All systems used to store and process the data are Federal Information Security Modernization Act (FISMA) compliant and undergo regular security review. Variables used solely for data cleaning purposes are also excluded. During initial processing, some standard checks are performed to ensure data are relatively clean, including ensuring that unique identifiers match across files, dropping variables with no data, identifying variables with unexpected values ( *i.e*. character values for an expected numeric variable), and identifying free-text variables that might benefit from standardization and/or translation. The study team is asked for clarification, revision, and input as needed. Creating ISA-based (Investigation, Study, Assay) files for loading often requires merging data files, and data conflicts can also be identified and fixed at that point.

To deal with the challenges of integrating distinct studies with highly heterogeneous data while providing user-friendly mechanisms to identify similar variables, we employ an ontology-based approach to generate a unified semantic framework as described in Zheng *et al.* (2016). Wherever possible, variables are mapped to existing terms drawn from Open Biological and Biomedical Ontologies (OBO) Foundry, which supports interoperable ontologies ( Smith *et al.,* 2007) through adhering to published principles. Necessary ontology terms are imported into the OBO Foundry registered VEuPathDB ontology, which is used as a single resource for all terms used in VEuPathDB resources. New ontology terms with VEuPathDB Internationalized Resource Identifiers (IRIs) are created as required. The use of ontologies to represent variables from different studies guides how data are loaded into relational databases and supports presentation of variables on the website to facilitate searching and analysis.

Once the data, ontology, and value mapping files are prepared, the data undergo processing to obfuscate dates to protect participant confidentiality. All dates for a given participant are consistently shifted forward or backward by 0–7 days according to a random number algorithm. While this obfuscation may introduce noise into longitudinal analysis—two events occurring on the same day for different participants will appear to have occurred 0–2 weeks apart—dates will still cluster within epidemiologically relevant timeframes for most analyses. All data are then transformed into an ISA-based format ( Sansone *et al.,* 2012) and loaded into a relational database based on the Genomics Unified Schema, version 4 (GUS4) ( Davidson *et al.,* 2001) running in an Oracle database management system (DBMS). Build database servers are located at the University of Pennsylvania and production instances are mirrored at the Universities of Pennsylvania and Georgia for redundancy purposes and to ensure uptime. All servers are housed in FISMA-compliant computational facilities, and industry standard backups of all data are performed.

Searches for each study are made available to users in an intuitive user interface (the “Search Wizard”), driven by a series of SQL queries against the GUS4 database (code available on GitHub, see *Software availability*). These searches vary depending on study design and record types. For example, in the longitudinal PRISM study, users can specifically retain or exclude observations occurring within a specified time relative to another observation through the “Related Observations” step in the Search Wizard (e.g. identify children diagnosed with febrile malaria at least twice within a six-month period). In the GEMS case-control study, users can compare cases to matching controls and choose whether to return data from the selected participants, matching cases/controls, or selected participants plus their matching cases/controls. Implementation of the strategies web development kit (WDK) ( Fischer *et al.,* 2011) allows users to construct even more complex queries using logical operators (union, intersection, subtraction) and to save and share search strategies.

Exploration applications for additional data visualization are created with Shiny, an open-source R package for building interactive web applications ( RStudio Inc, 2019). The applications are hosted on the website via the Shiny Server Open Source software. SQL queries against the Oracle database identify all variables in the study and their format, which informs which variables appear as options to plot, how to build a custom dichotomous variable ( *i.e.* hemoglobin <=10 mg/dL vs. hemoglobin >10 mg/dL), and how the data are plotted within the applications.

Studies are reviewed by ClinEpiDB staff for quality control and made accessible to primary data providers using a protected internal website to ensure data accuracy and query functionality. Data are only scheduled for public release following data provider approval. Updates to the database are released every two months and can include new studies, features, and/or software updates.

### Operation

ClinEpiDB can be accessed via any web browser at https://clinepidb.org. User support is available via the “Contact Us” link and tutorials are accessible via the “Community” drop-down menu at the top of each web page.

**Figure 2.**
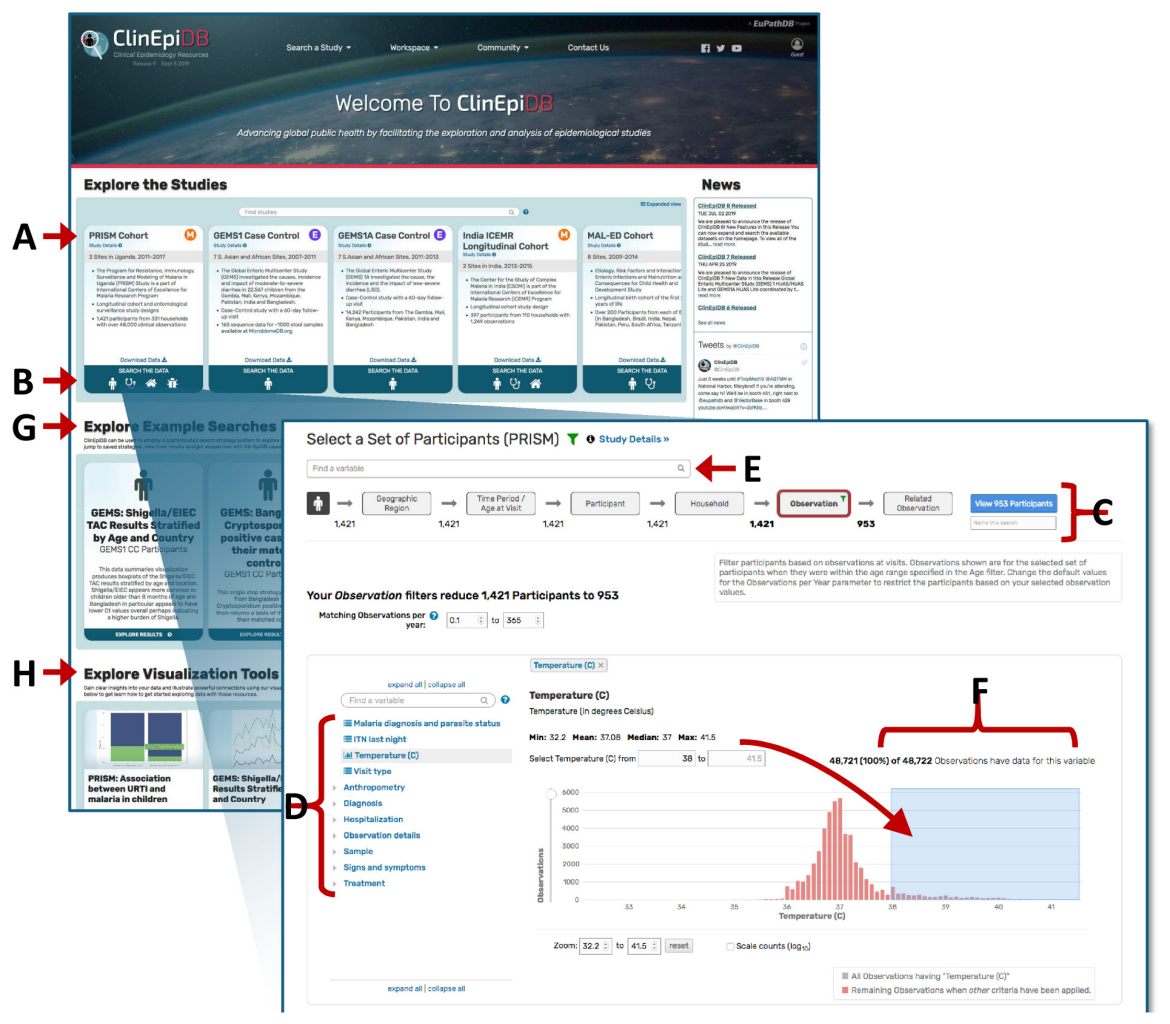
Using the Search Wizard to explore variables. (A) Clicking a card study name opens a study page. (B) Clicking on a card search icon initiates a search. (C) The Search Wizard categorizes the variables into discrete steps. The grey buttons let users move between steps. (D) The variable tree contains all variables within that step of the Search Wizard. To subset the data, users can open a variable from this tree. (E) The “Find a variable” search bar searches for variables based on variable names and values across all Search Wizard steps. (F) Continuous data are displayed as a histogram and can be constrained by typing the exact range of values or clicking and dragging the mouse across the range of interest. (G) Clicking cards underneath “Explore Example Searches” opens up examples of searches conducted using the datasets indicated. These searches can be edited. (H) Clicking cards underneath “Explore Visualization Tools” opens up examples of how the exploration applications can be used.

***Study pages.*** Clicking a study name on a card on the ClinEpiDB homepage ( Figure 2A) or under the “Search a Study” drop-down menu brings up the study page, which provides a description of that particular study’s goals and objectives, methodology, investigators, and links to associated publications. This page also provides links to data collection forms and data dictionaries, which detail variable definitions, allowed values, skip patterns, etc. For studies that require permission to download data, the study page also incorporates a table listing individuals who have been granted access to the data and their brief stated purpose of use.

***Search strategies.*** ClinEpiDB permits users to execute searches on epidemiological data sets. Depending on available study data, up to four search types are currently supported that identify Households, Participants, Observations, or Entomology Collections of interest ( Table 2). For example, surveillance studies with just a single observation per participant offer only a participant search (e.g. “How many participants presented with both a fever and cough”). Studies with multiple participants from the same household will have household searches as well (e.g. “Which households contained children with asymptomatic parasitemia”). Longitudinal studies permit observation-level searches (e.g. “Identify all observations of children with malaria from houses with unscreened windows”). Within each search, users can subset the data based on any of the variables available through the “Search Wizard”, explore associations between variables, and return an interactive table of selected data.

**Table 2.** Search types currently available in ClinEpiDB.

Search type	Default steps available via the Search Wizard	Results Table format	Results Table variables
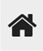 **Household**	Household Participant Observation	One row per household observation (multiple rows per household if household data was collected longitudinally)	Household-level variables relating to geographic location, dwelling characteristics, socioeconomic status, etc.
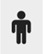 **Participant**	Household Participant Observation	One row per participant	Participant-level variables relating to demographics, enrollment, data summaries, etc. May also include upstream (household-level) variables.
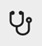 **Observation**	Household Participant Observation	One row per observation (multiple rows per participant if data was collected longitudinally)	Observation-level variables relating to anthropometry, symptoms, laboratory test results, treatment, etc. May also include upstream (household- and participant-level) variables.
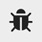 **Entomology**	Household Entomology collection	One row per entomology collection (multiple rows per household if collections were done in multiple rooms or longitudinally)	Entomology variables relating to mosquito counts and species. May also include upstream (household-level) variables.

**Figure 3.**
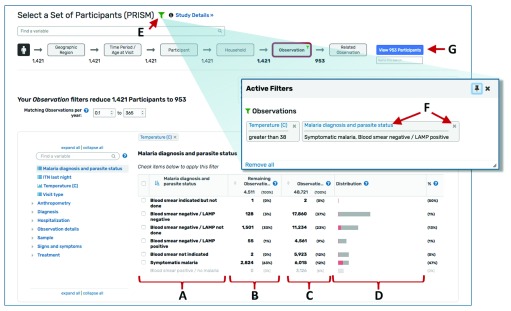
Adding, editing, and removing filters. Categorical data are displayed in a table and (A) can be selected via check boxes next to the values. (B) The “Remaining” column indicates the data remaining given all other data selections (including selections in upstream steps), while the (C) Observations column indicates the total counts for all data. (D) For both continuous and categorical variables, data that meet the filter criteria (“remain”) are shown in red on the distribution graph while data that do not meet the filter criteria are shown in grey. (E) Clicking the green filter icon brings up a box that lists all applied filters. (F) Users can click the blue link to edit a filter or the “x” to remove it. (G) The blue button takes the user to the results page.

From the home page, clicking a search icon on a study card initiates a search ( Figure 2B). The “Search Wizard” at the top of the page ( Figure 2C) categorizes the data, providing a step-wise approach to selecting data. On the left-hand side of the page, the variable tree presents all variables within that step of the Search Wizard ( Figure 2D), while the search bar at the top of the page allows users to search for variables across all Search Wizard steps ( Figure 2E). To subset data, users click on a variable of interest (e.g. “Temperature (C)”) and specify desired values. Continuous data are displayed as a histogram and can be selected by typing in a specific range or by clicking and dragging the cursor across the range of interest ( Figure 2F). Categorical data (e.g. “Malaria diagnosis and parasite status”) are displayed in a table and can be selected via the adjacent check boxes ( Figure 3A).

As data are selected, the data available for other variables in that Search Wizard step and any downstream steps are dynamically updated so the user can visualize the impact of their selection(s) on other variables. The “Remaining” column in the variable tables indicates the data remaining given all upstream filters ( Figure 3B), while the column to the immediate right indicates the total counts ( Figure 3C). For both continuous and categorical variables, data meeting upstream selection criteria are shown in red on the distribution graph while data excluded by the selection criteria are shown in grey ( Figure 3D). Selections can be reviewed, edited, or removed by clicking the green filter icon ( Figure 3E) and then clicking the blue link to edit selections for a variable or the “X” to remove it ( Figure 3F). Combined with data visualization through bar charts and histograms, this ability to conveniently add, edit, and remove filters makes it possible to rapidly assess the structure of the data and potential associations between variables of interest.

New users wanting to get a sense of what types of searches are possible can choose to view and edit publicly available searches under the “Explore Example Searches” section of the homepage ( Figure 2G).

**Figure 4.**
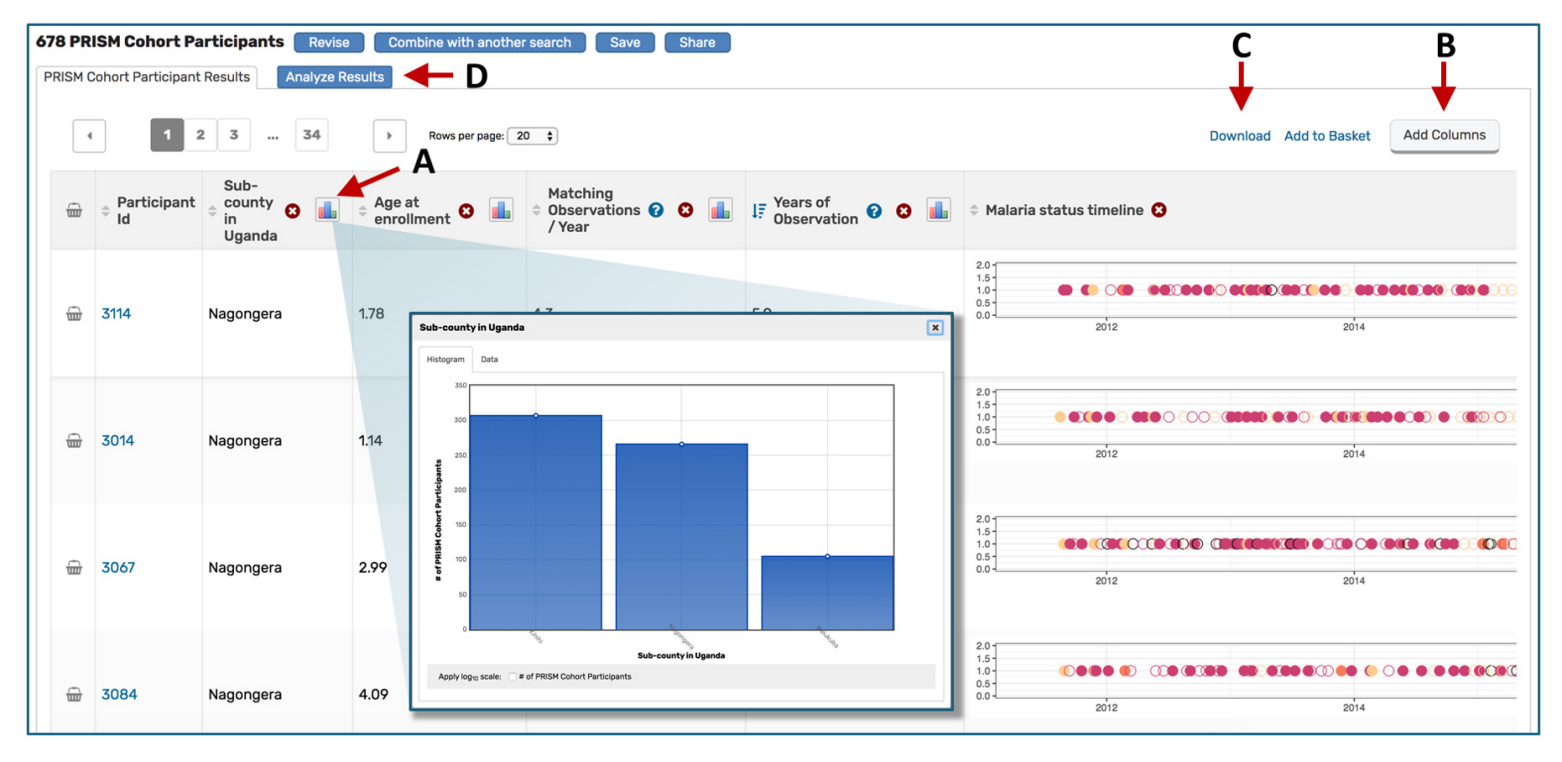
Using the Results Page. (A) Clicking a histogram icon opens a pop up showing the distribution of data for that variable. (B) The “Add columns” button allows users to change which variables are shown in the table. (C) The “Download” link directs users to a page where they can choose which variables to download. The data subset is based on the selections applied in the Search Wizard. (D) The “Analyze Results” tab leads to a suite of applications for further data visualization.

***Results page and exploration apps.*** Data selected as described above are displayed on the Results Page ( Figure 4) when the user clicks the blue button at the right-hand terminus of the Search Wizard ( Figure 3G). The selected data are displayed as a table ( Figure 4) and may be passed to a suite of web applications for additional visualization and analysis. Variables available as columns are based on the type of search performed ( Table 2). Histogram icons in the column headers allow users to assess the distribution of the subset of data for that variable ( Figure 4A); links in the top right corner allow users to add additional variable columns ( Figure 4B) or download the selected data ( Figure 4C).

The “Analyze Results” tab ( Figure 4D) leads to a suite of web applications. Three applications are currently available in ClinEpiDB: Distributions, Contingency Tables, and Data Summaries (also accessible under “Explore Visualization Tools” on the homepage; Figure 2H). The Distributions application shows the distribution of any variable in the data set and allows stratification based on other variables. The Contingency Table application generates a 2 × 2 contingency table for two selected variables and calculates a p-value, odds ratio, and relative risk, enabling assessment of associations (note that these statistics should be interpreted with caution as they do not control for confounding or other forms of bias). The Data Summaries application plots a variable of interest over time for longitudinal studies or two variables of interest against each other for non-longitudinal studies. For each app, users can toggle between tabs to define plot parameters, view summary statistics, display a plot grid or individual plots, and obtain help. Drop-down menus allow users to specify which variables to graph and whether to stratify data based on additional variables. Updating parameters automatically regenerates all statistics and plots. By default, the applications utilize the entire study data set, but users may choose to examine data selected in the Search Wizard by stratifying based on search results. The plots may be downloaded, but we encourage users to use the applications primarily for initial data exploration and to make their own graphs for presentations and publications after careful execution of a statistically robust, thorough data analysis, considering any potential biases and statistical assumptions.

***Data downloads.*** Data in ClinEpiDB may be downloaded in two ways. Clicking the “Download” link on the Results Page ( Figure 4C) allows users to customize downloads, specifying which variables to retrieve based on the search type (see Table 2). All other variables can be downloaded, and data can be linked across files via observation, participant, and household IDs. Users may specify .txt or .csv formats, both of which can be consumed by most modern data analysis tools. Users can also download the entire data set via the “Download Data” link on the homepage study card and the study page. An ontology term association file links variables to their original study labels so users can reference study data collection forms and data dictionaries to learn more about each variable. Variables are also mapped to ontology terms via Internationalized Resource Identifiers (IRIs) which are included in each column header of the download file ( Ong *et al.,* 2017). Following OBO Foundry principles, the terms are reused or requested from existing ontologies when possible but placeholder terms are also created as needed. Once defined, the terms are made public in the VEuPathDB application ontology along with imported terms from other ontologies and are searchable on Ontobee.

### Accessibility of datasets

ClinEpiDB is committed to making epidemiologic data sets accessible to global research and biomedical communities while protecting the rights of study participants and data providers. Prior to viewing the website for the first time, users are required to agree to a Data Access and Use Policy outlining expectations regarding data use, protection of participant privacy, and acknowledgement of data providers and ClinEpiDB.

Some studies require data access restrictions at the data provider’s discretion. There are five access levels data providers can choose from that differ in their requirements for users to view aggregate versus disaggregate data ( Table 3). Aggregate data are accessible in the Search Wizard and through the exploration applications, while disaggregate data can be found on the results page, individual record pages, and in the download files. Except for studies classified as private, which require approval to see any data, users can see variables and aggregate data for all studies. Note that on occasion, variables that are potential indirect identifiers are loaded into the database but not displayed on the website. These variables are available for download to individuals with access approval.

**Table 3.** Data access restriction levels.

Access level	Description
Public	No access restrictions. Users can view and download all data as a “Guest” without logging in.
Controlled	Users can view data in the Search Wizard, in exploration applications, and view the results pages and recordpages as a “Guest” without logging in, but must obtain approval from the data providers to download data.
Limited	Users can view data in the Search Wizard and exploration applications as a “Guest” without logging in, but must log in with a registered account to view more than 20 rows of data on the results page or view individual record pages. Users must obtain approval from the data providers to download data.
Protected	Users can view data in the Search Wizard and exploration applications as a “Guest” without logging in, but must obtain approval from the data providers to view more than 20 rows of data on the results page, view individual record pages, or download data.
Private	Users must request and obtain approval to access any aspect of the data.

When a user reaches a restricted section of the website, they are automatically prompted to either log in with a ClinEpiDB account or log in and submit a data access request, depending on the access restrictions. The data access request form requires the purpose for which the requested data will be used, whether the requester has been in contact with the study team, hypotheses and/or research questions, analysis plan, and planned dissemination of results. The request is then sent to data providers for approval. Users are contacted within a few days with any conflicts that are identified or with notification of approval. Once approved, they may view and download that study’s data at any point by logging into their ClinEpiDB account. To ensure transparency and promote collaboration within the wider scientific community, the requestor’s name, organization, request date, and indicated purpose appear publicly on the corresponding study page once approved.

## Use cases

ClinEpiDB provides a powerful web-based platform that enables the research community to easily access and explore clinical epidemiological data for primary and secondary use via an intuitive point-and-click interface, maximizing potential for generating new, data-driven hypotheses and promoting collaborations between researchers.

Two examples focusing on the PRISM data ( Dorsey *et al.,* 2018), the first study released on ClinEpiDB, illustrate how the website can be used by potential collaborators looking for samples and analysts looking for data to inform modeling. In the first instance, a collaborator interested in accessing and analyzing peripheral blood mononuclear cell (PBMC) samples from timepoints close to when a participant was diagnosed with malaria was able to identify the appropriate samples themselves using ClinEpiDB and begin generating preliminary data. By initiating an observation search and setting “Sample type” to “PBMC”, they were able to determine that 5295 PBMC samples were collected during the study. Next, by going to the “Related Observation” step in the Search Wizard, opting to “ *Keep* Observations within *0–10* days *after* the Related Observation specified below” and selecting data where “Malaria diagnosis and parasite status” was “Symptomatic malaria”, they were able to identify 130 PBMC samples collected within 10 days of a malaria diagnosis (see saved strategy). In a second example, a student was able to examine the data using ClinEpiDB to determine a difference in the percent of malaria-attributable fever based on whether fever was self-reported or measured. By running an observation search and limiting “Temperature (C)” to greater than or equal to 38 then looking at where “Asexual Plasmodium parasites present, by microscopy” was positive, they found that 2824 of 4508 observations of measured fever (62.6%) could be attributed to malaria. In contrast, looking at observations where “Subjective fever” was reported and where “Asexual Plasmodium parasites present, by microscopy” was positive revealed that 6006 of 15,228 observations (39.4%) of self-reported fever could be attributed to malaria. They planned to use those statistics to adjust a model that uses data on self-reported fever. 

An additional hypothetical example highlights how users might explore data in ClinEpiDB before deciding to submit a data access request to download the data for further analysis. A user might be interested in re-analyzing risk factors for rotavirus infection and disease in children based on new molecular diagnostics testing for enteropathogens in MAL-ED stool samples ( Platts-Mills *et al.,* 2018; Spiro *et al.,* 2019) instead of ELISAs, as done previously ( Mohan *et al.,* 2017). To quickly determine if secondary analysis is worthwhile, the user would perform an Observation-level search of MAL-ED, choosing the Observation step from the Search Wizard, and selecting the entire range of Cycle threshold (Ct) values under “Rotavirus Ct value, by TAC result” to limit analysis to samples that underwent TAC testing for rotavirus. Setting “Stool type” to “Diarrhea” reveals that 6745 diarrheal stool samples were tested for rotavirus using TAC. By navigating back to “Rotavirus CT value, by TAC result” and setting the range of Ct values to “<31.7” (the TAC cut-off for rotavirus defined in Platts-Mills *et al*. (2018) and then returning to “Stool type”, the user would observe that 568 (8.4%) of 6745 diarrheal stool samples were positive for rotavirus using TAC (see saved strategy). Substituting “Rotavirus, by ELISA” for “Rotavirus CT value, by TAC result,” the user would then discover that 535 (5.7%) of 9301 diarrheal stool samples were positive for rotavirus by ELISA, consistent with the report by Mohan *et al*. (2017) (see saved strategy). Such study exploration enables rapid evaluation of whether or not a robust statistical reanalysis using the more sensitive molecular diagnostic data would be feasible.

## Conclusions

Journals and funders increasingly require that data be made publicly available ( National Institutes of Health, 2003; The Wellcome Trust, 2011), but data hidden in supplementary data files or stored in data repositories are often difficult to locate, interpret, or use by those not actively engaged in the study. ClinEpiDB strives to follow FAIR Guiding Principles ( Wilkinson *et al.,* 2016) by creating resources, tools, vocabularies, and infrastructure that supports third-party discovery and reuse of primary epidemiological research data. Studies loaded into ClinEpiDB are provided with stable, unique identifiers, making them “Findable.” An intuitive interface and visualization tools allow users to see and directly query the data, lowering the barrier for exploratory data analysis. While these tools are not a substitute for rigorous, controlled statistical analyses, data can be downloaded in common machine-readable formats for robust analysis, making it more “Accessible.” The implementation of standardized, publicly available ontologies makes the data more “Interoperable.” Even when similar variables in different studies map to distinct ontology terms, the display labels, definitions, and position of the variable in the variable tree provide useful information that allow users to generate similar queries for different studies. Study pages are always public and provide context that makes the data more “Reusable.”

As ClinEpiDB continues to be developed, users can expect to see the release of additional studies focusing on malaria, enteric disease, respiratory disease, and more. Additional long-term development plans include strengthening and expanding data visualization and exploration tools. Epidemiologic data loaded into ClinEpiDB is currently separate from genomic data available via other EuPathDB resources such as PlasmoDB ( Bahl *et al.,* 2003) or MicrobiomeDB ( Oliveira *et al.,* 2018), but the use of common infrastructure creates the possibility of queries across currently disparate resources, facilitating additional secondary data use.

In summary, the ClinEpiDB platform promotes access and interrogation of complex epidemiological studies loaded in the database through a user interface that enables visualization of and interaction with all data within a study. Regular release of additional studies along with new features is expected to further support secondary data use. Similar to what has been achieved through the EuPathDB websites, production of ClinEpiDB will help maximize the impact of the epidemiology studies that are loaded and abbreviate time to discovery while stimulating productive collaborations between research groups.

## Data availability

All data underlying the results are available as part of the article and no additional source data are required.

## Software availability

Infrastructure description of repositories available from: https://eupathdb.org/eupathdb/wdkCustomization/jsp/questions/XmlQuestions.Infrastructure.jsp

Source code available from: https://github.com/VEuPathDB

Archived source code at the time of publication: https://doi.org/10.5281/zenodo.3522209

License: GNU Library General Public License v2

All GitHub repositories are publicly available except for ClinEpiPresenters, since this repository may contain information on studies that are not yet ready for release. The archived source code includes a version of this repository where information has been redacted for studies that have not yet been released.
