# Supplementary material for: ClinEpiDB: an open-access clinical epidemiology database resource encouraging online exploration of complex studies
Source: Gates Open Res. 2020 Apr 6;3:1661. Originally published 2019 Nov 25. [Version 2] doi: 10.12688/gatesopenres.13087.2 (PMC6993508; doi:10.12688/gatesopenres.13087.2)
Supplement: Peer review history

# Peer review: Reviewer response for version 2

**DOI:** 10.21956/gatesopenres.14285.r28746

**Published:** 2020-04-06

**Authors:** Nicki Tiffin

**Affiliations:** 1 Computational Biology Division, Integrative Biomedical Sciences, Wellcome Centre for Infectious Disease Research in Africa, Centre for Infectious Disease Epidemiology and Research, University of Cape Town, Cape Town, South Africa

Thank you to the authors for their considered and informative responses to the points I raised. I look forward to seeing further developments with this useful and carefully compiled resource going forward.

I confirm that I have read this submission and believe that I have an appropriate level of expertise to confirm that it is of an acceptable scientific standard.

# Peer review: Reviewer response for version 1

**DOI:** 10.21956/gatesopenres.14232.r28350

**Published:** 2020-01-27

**Authors:** Nicki Tiffin

**Affiliations:** 1 Computational Biology Division, Integrative Biomedical Sciences, Wellcome Centre for Infectious Disease Research in Africa, Centre for Infectious Disease Epidemiology and Research, University of Cape Town, Cape Town, South Africa

Thank you for the invitation to review this interesting, comprehensive and accessible paper. This data harmonisation and integration platform provides a well-tailored and intuitive front end that facilitates exploration of existing epidemiological and clinical datasets under a tiered access model. Curation of the data sets prior to making them generally available, as well as harmonisation of data elements to existing data standards, is undertaken to ensure data quality. Great care has been taken to ensure participant confidentiality, with some basic perturbation and obfuscation steps undertaken to prevent re-identification of participants from longitudinal or complex data. This resource can provide datasets for further analysis and secondary studies, and can also assist researchers in ensuring their data are share-able when publishing in journals requiring this; but will also provide options for access control that can reflect participants’ preferences and ethical approvals for the onward use of their data.

 I explored the data for the PRISM study. The query builder and filters are intuitive and informative, and the total data remaining that are shown with each filtering step are helpful. The information provided for the study is also clearly displayed and easy to access.

 I have a few minor points that I think might improve the resource, and/or provide some useful information about it. 

- This paper is presented more like a user guide describing comprehensively the different ways in which an end user can access, query and visualise datasets, or download them. I think it would be informative to provide a paragraph describing the specifications for the back end of the system, such as where the data are stored (which also informs under what jurisdiction the data fall), what back-end databasing system is used, what are the security and governance implementations of the database (including back-ups, access control systems etc). 
- (A suggestion for future work): Particularly in the Observations section of the data, it was unclear to me whether clinical coding is used for treatments, laboratory tests, diagnosis etc? This might be useful addition to make in the future to ensure standardisation of data capture, for example mapping to ICD10/ICD11, ATC or Loinc codes. 

Whilst the obfuscation and perturbation of data undertaken is commendable, I think there are a few extra steps that might easily be implemented: 

- Age in years to two decimal places is very specific and could assist re-identification of an individual. Perhaps a sliding scale could be used instead, something like: age in days for category 0-2 weeks, age in weeks for up to 2 months, age in months up to 2 yrs, and age in years (integers) thereafter. At some point, age to two decimal places is not any more meaningful than age as an integer, but it provides an additional layer to prevent re-identification.
- Similarly, measurements that have many decimal places could conceivably be used to re-identify individuals, and number of decimal places can often be restricted without altering meaning (For example, in our own work, birth weight in kg is recorded to four decimal places, and given approximate time of birth, location or other similar filter, could potentially be used to re-identify individuals. Restricting birth weight to one or two decimal places does not alter epidemiological value of the measurement). For a set of longitudinal measurements, the risk of re-identification becomes even higher.
- As the same integer is used per individual to offset the dates in the dataset, I wonder if 7 is a little low as an offset limit? Increasing this limit to, for example, 30 or 50 should not alter epidemiological validity whilst significantly reducing risk of re-identification. I am interested in the choice of one week - so am satisfying my own curiosity, too, in asking this question rather than proposing a change in the current limit.
- As a secondary user of data, I like to see what information the participants received about how their data are used, and what form they have signed for the use of those data: I like to be sure that the participant knows how their data are being used, and approve of that use (and that I am not contravening their approvals). It would be useful and appropriately transparent if the participant information document and informed consent template could be provided along with the CRFs and data dictionaries, as well as the ethics approval letter/document. Perhaps they are provided somewhere else and I have missed it, but this seems an intuitive place to be able to access these documents, to ensure my intended secondary use is in line with participant consents. This would also permit the downstream data consumers to take personal responsibility for deciding on what is appropriate, consented and ethical re-use of the data, in addition to such decisions taken by data submitters and curators. 
- It is unclear to me why the curators at ClinEpiDB receive identified data, and do the de-identification – if I have understood this correctly. Would it be preferable if data submitters were to replace personal and identifying information with anonymised IDs prior to submitting their datasets? 
- Whilst I appreciate that this is a Methods/DB paper, my interest is immediately raised as to what participants think of this use of their data; and I do believe this should be a consideration for any resource that provides open access to sensitive participant data. I think it would be an appropriate and responsible addition to the paper to add just a short sentence to the ethics section to describe what community engagement programs were undertaken to understand acceptability of this data-use for the participants for the prototype research program. When accepting data sets to the platform, perhaps community engagement information could be some of the metadata that are collected and presented on the study page, wherever available.

I confirm that I have read this submission and believe that I have an appropriate level of expertise to confirm that it is of an acceptable scientific standard.

# Response

**Published:** 2020-03-20

**Authors:** Sheena Tomko

*Thank you for the invitation to review this interesting, comprehensive and accessible paper. This data harmonisation and integration platform provides a well-tailored and intuitive front end that facilitates exploration of existing epidemiological and clinical datasets under a tiered access model. Curation of the data sets prior to making them generally available, as well as harmonisation of data elements to existing data standards, is undertaken to ensure data quality. Great care has been taken to ensure participant confidentiality, with some basic perturbation and obfuscation steps undertaken to prevent re-identification of participants from longitudinal or complex data. This resource can provide datasets for further analysis and secondary studies, and can also assist researchers in ensuring their data are share-able when publishing in journals requiring this; but will also provide options for access control that can reflect participants’ preferences and ethical approvals for the onward use of their data.*

*I explored the data for the PRISM study. The query builder and filters are intuitive and informative, and the total data remaining that are shown with each filtering step are helpful. The information provided for the study is also clearly displayed and easy to access.*

 We thank the reviewer for her time and thoughtful critiques. We have done our best to address them in the manuscript and this response.

*I have a few minor points that I think might improve the resource, and/or provide some useful information about it.*

*·* *This paper is presented more like a user guide describing comprehensively the different ways in which an end user can access, query and visualise datasets, or download them. I think it would be informative to provide a paragraph describing the specifications for the back end of the system, such as where the data are stored (which also informs under what jurisdiction the data fall), what back-end databasing system is used, what are the security and governance implementations of the database (including back-ups, access control systems etc).*

 Thank you for the suggestion. We have updated the manuscript implementation section to provide general information on our servers and the fact that they are FISMA-compliant. 

 · *(A suggestion for future work): Particularly in the Observations section of the data, it was unclear to me whether clinical coding is used for treatments, laboratory tests, diagnosis etc? This might be useful addition to make in the future to ensure standardisation of data capture, for example mapping to ICD10/ICD11, ATC or Loinc codes.*

 So far the data we have received from data providers and integrated has not included any standardized clinical coding, and we have not attempted to add it. However, it should be noted that our ontology approach to representing the data enables us to relate clinical information as well as other types of information like demographics, healthcare utilization and attitudes, or environmental risk variables. Standard diagnoses-oriented ICD codes will not capture the breadth of data collected for research but we are actively exploring how we can further annotate or integrate existing standardized terminologies.

*Whilst the obfuscation and perturbation of data undertaken is commendable, I think there are a few extra steps that might easily be implemented:*

*·* *Age in years to two decimal places is very specific and could assist re-identification of an individual. Perhaps a sliding scale could be used instead, something like: age in days for category 0-2 weeks, age in weeks for up to 2 months, age in months up to 2 yrs, and age in years (integers) thereafter. At some point, age to two decimal places is not any more meaningful than age as an integer, but it provides an additional layer to prevent re-identification.*

*·* *Similarly, measurements that have many decimal places could conceivably be used to re-identify individuals, and number of decimal places can often be restricted without altering meaning (For example, in our own work, birth weight in kg is recorded to four decimal places, and given approximate time of birth, location or other similar filter, could potentially be used to re-identify individuals. Restricting birth weight to one or two decimal places does not alter epidemiological value of the measurement). For a set of longitudinal measurements, the risk of re-identification becomes even higher.*

 We appreciate the concern regarding participant re-identification. One approach that we take to reduce the risk of re-identification is to obfuscate all dates recorded in the study, as you allude to. A second approach is to load variables so that they are available in the download files, but are unavailable to the general public on the website. We have used this with variables like date of birth, location, variables capturing the participant’s history of hospitalization, *etc.* so that only researchers approved to download the data can access this information.

 In general, most of the measurement data we receive are 0-2 decimal places, but your point is well taken, and we will ask data providers to consider whether it is acceptable to restrict the number of decimal places. Similarly, for age, we will ask the study team if it’s appropriate to reduce the number of significant figures, particularly for older children and adults.

*·* *As the same integer is used per individual to offset the dates in the dataset, I wonder if 7 is a little low as an offset limit? Increasing this limit to, for example, 30 or 50 should not alter epidemiological validity whilst significantly reducing risk of re-identification. I am interested in the choice of one week - so am satisfying my own curiosity, too, in asking this question rather than proposing a change in the current limit.*

 Thank you for the question. The offset is up to 7 days in either direction, so events happening to different people on the same day will appear to have occurred 0-2 weeks apart from each other. This was chosen so dates will still cluster within epidemiologically relevant timeframes for many infectious diseases. In areas with seasonal malaria transmission, for example, a higher offset of up to 25 days in either direction might push enough events of malaria outside the normal season that it changes the results of an exploratory analysis. With the 7 day offset, we hope to strike a balance that protects participants' identities while still allowing for meaningful, hypothesis generating analyses online. For specialized analyses requiring exact dates, we recommend users request the original data files.

*·* *As a secondary user of data, I like to see what information the participants received about how their data are used, and what form they have signed for the use of those data: I like to be sure that the participant knows how their data are being used, and approve of that use (and that I am not contravening their approvals). It would be useful and appropriately transparent if the participant information document and informed consent template could be provided along with the CRFs and data dictionaries, as well as the ethics approval letter/document. Perhaps they are provided somewhere else and I have missed it, but this seems an intuitive place to be able to access these documents, to ensure my intended secondary use is in line with participant consents. This would also permit the downstream data consumers to take personal responsibility for deciding on what is appropriate, consented and ethical re-use of the data, in addition to such decisions taken by data submitters and curators.*

 We appreciate the suggestion. Going forward, we will ask the study team to provide the informed consent template, participant information document, and ethics approval letter.

*·* *It is unclear to me why the curators at ClinEpiDB receive identified data, and do the de-identification – if I have understood this correctly. Would it be preferable if data submitters were to replace personal and identifying information with anonymised IDs prior to submitting their datasets?*

 While we always recommend that the study team remove identifying information prior to providing us the files, on rare occasions, we still receive that information. In these cases, we remove the identifying information before storing the files on our servers to protect participant confidentiality, and that information is never loaded into the Oracle databases serving our websites.

*·* *Whilst I appreciate that this is a Methods/DB paper, my interest is immediately raised as to what participants think of this use of their data; and I do believe this should be a consideration for any resource that provides open access to sensitive participant data. I think it would be an appropriate and responsible addition to the paper to add just a short sentence to the ethics section to describe what community engagement programs were undertaken to understand acceptability of this data-use for the participants for the prototype research program. When accepting data sets to the platform, perhaps community engagement information could be some of the metadata that are collected and presented on the study page, wherever available.*

 We appreciate the suggestion and have added a statement to the manuscript indicating that community engagement programs assessing acceptability of data re-use through the platform have not yet been performed. Going forward, we will ask study teams if they have done any assessments of community attitudes towards data reuse and include that information on the study page, if available.

# Peer review: Reviewer response for version 1

**DOI:** 10.21956/gatesopenres.14232.r28307

**Published:** 2020-01-02

**Authors:** Faith Osier, Kennedy Mwai

**Affiliations:** 1 KEMRI-Wellcome Trust Research Programme, CGMR-C, Kenya Medical Research Institute (KEMRI), Kilifi, Kenya; 2 Kemri-Wellcome Trust Research Programme, Kenya Medical Research Institute (KEMRI), Kilifi, Kenya

In general, the paper is interesting, well written paper describing the use of a web-based approach to reduce the barrier to improve data access in an innovative and promising approach manner. The authors present a point-and-click platform that not only allows data accessibility but also provides visualization options and different approaches to the sub-setting of the data. The authors have in detail explained the operation, search strategies and usability of the software in detail, assisted by the use of diagrams and screenshots. The software in this paper clearly shows the usability and operation of the software. The following minor revisions are suggested.

 Minor Revisions:

 The authors should make it clear what the added value of this tool is over existing repositories. Different repositories that allow data to be Findable, Accessible, Interoperable and Reusable (FAIR). They report that are in existence and the authors have mentioned a few of them. ClinEPiDB provides extra features to assist in translational research and secondary data discovery. However, the authors have not clearly explained the underlying principle for developing this software. Is this all? Could this be more illustrative? For example, the authors also mention that ClinEPiDB was developed with the landscape of CHAMPS repository. A clear justification of what ClinEPiDB, as a software adds over CHAMPS repository, would be of importance for the readers. How does ClinEPiDB stand out. This will help us to have an idea of what this software adds to the data sharing repositories.  

 The authors mention the technicalities packages applied utilized in the software tool but do not not clearly expand in how these are connected. For example Fig 1 shows how different file types are connected, but no detail is provided on exactly what is done with different variable formats, especially when these are processed in different software. A flow chart linking these software would be helpful. For example, from the repositories, it was not clear from the repositories whether Shiny runs independently as an add on or its part of a repository.

 Additionally, the pre-processing steps that must happen before bits of data are merged are not explained and are important. How could a user get a sense of the quality of the data that’s uploaded onto the platform. Provision of the processing steps before data were uploaded would be helpful.

 The shifting of dates for a given participant is important for data security and privacy. Could the authors discuss the likely impact of this on functions like how can this affect maybe the fitting of longitudinal/time series models?

 Could the authors clarify and broaden the range of study designs that can or cannot be utilized in ClinEpiDB.  

 One of the advantages I have observed in ClinEpiDB is the ability to allow users to integrate and visualize different studies. This integration is important to allow analysis of data from multiple sites. However, although the authors promote the use of an ontology-based approach {Open Biological and Biomedical Ontologies}, they do not outline the principles underlying it. Explaining how to deal with integrating distinct studies, mention the ontology-based approach {Open Biological and Biomedical Ontologies} but lack in brief/detail how this ontology works. Does it use a single, hybrid or multiple ontology for instance.

 The authors include a visualization add on using in a point in click software package which that is very friendly to use. During the use of the software and using Figure 4 as an example, a download button is not available for the graphs produced. Additionally, for one the images produced with plotly on the shiny visuals, only the default download option is available. Maybe perhaps a high res image download option or a reproducible code would be useful to allow one to export high quality graphs for publications or presentations.

 The help page would be more useful as a community forum such that a body of users builds up that can be monitored by the administrator. In this way, users could learn from the queries and responses from other users/administrators and this could save a lot of time for example when similar issues are raised repeatedly.

 The authors should consider making it a requirement that any publications arising out of data that is generated using this platform is fully open access. This should apply not only to the data, but to the code used to subset and generate the data to allow full transparency and the ability of other users to reproduce analyses and figures.

We confirm that we have read this submission and believe that we have an appropriate level of expertise to confirm that it is of an acceptable scientific standard.

# Response

**Published:** 2020-03-20

**Authors:** Sheena Tomko

*In general, the paper is interesting, well written paper describing the use of a web-based approach to reduce the barrier to improve data access in an innovative and promising approach manner. The authors present a point-and-click platform that not only allows data accessibility but also provides visualization options and different approaches to the sub-setting of the data. The authors have in detail explained the operation, search strategies and usability of the software in detail, assisted by the use of diagrams and screenshots. The software in this paper clearly shows the usability and operation of the software. The following minor revisions are suggested.*

 We thank the reviewers for their thoughtful review of our manuscript. We appreciate the points that were raised and have done our best to incorporate them into the updated manuscript.

*Minor Revisions:*

*The authors should make it clear what the added value of this tool is over existing repositories. Different repositories that allow data to be Findable, Accessible, Interoperable and Reusable (FAIR). They report that are in existence and the authors have mentioned a few of them. ClinEPiDB provides extra features to assist in translational research and secondary data discovery. However, the authors have not clearly explained the underlying principle for developing this software. Is this all? Could this be more illustrative? For example, the authors also mention that ClinEPiDB was developed with the landscape of CHAMPS repository. A clear justification of what ClinEPiDB, as a software adds over CHAMPS repository, would be of importance for the readers. How does ClinEPiDB stand out. This will help us to have an idea of what this software adds to the data sharing repositories.* 

 Thank you for the suggestion. We have updated the manuscript to more clearly indicate how ClinEpiDB adds value by mapping variables to common ontologies, creating a unified semantic framework capable of incorporating studies with different disease foci. This framework underpins the website, which encourages investigators to explore data online through interactive tables, graphs, and a simple, visual query interface, reducing the time and effort required to determine what data are available and if they are worth further analysis compared to repositories where subject level data is often only available as data files and investigators may need to submit an access request form to even see what variables are included. ClinEpiDB allows users to safely see and manipulate aggregations of subject level data to explore study data in ways other repositories do not.

*The authors mention the technicalities packages applied utilized in the software tool but do not not clearly expand in how these are connected. For example Fig 1 shows how different file types are connected, but no detail is provided on exactly what is done with different variable formats, especially when these are processed in different software. A flow chart linking these software would be helpful. For example, from the repositories, it was not clear from the repositories whether Shiny runs independently as an add on or its part of a repository.*

 Variable formats change over the course of data processing and loading. During initial processing, data files are imported into R, and R scripts are used to generate the variableMap and valueMap files. If data files are provided in .csv or .txt formats, those files are used to create the merged ISA-based files for loading, without changes to the variable formats. Files provided in other formats such as .dta, are saved as .txt files using R, so all variable formats are converted to string. During our data loading process, the value mapping is applied, so variables that originally contained coded categorical values, which might appear as numeric in the original data files, will now have string values. Additionally, numeric variables that originally contained special characters to indicate missing or not applicable data will have the special characters removed so they only contain numeric values. A script then automatically identifies the data format for each variable - string, number, or date - before loading into the Oracle database. The user interface of the website displays the data based on the format - strings are displayed as tables, while numbers or dates with more than 10 values are displayed as histograms.

             With regards to Shiny, we have updated the manuscript to reduce the amount of attention on Shiny in particular since it is one package of many that we use in making our website. We have accomplished this by changing the references to the applications from “Shiny apps” to “exploration applications”. We have also corrected our oversight in not providing enough detail about how the applications function within the website by adding to the implementation section. Briefly, the code for the analysis applications lies primarily within the ClinEpiWebsite repository, and the applications are hosted on the website via the Shiny Server Open Source software. SQL queries against the Oracle database identify all variables in the study and their format, which informs which variables appear as options to plot, how to build a custom dichotomous variable (i.e. hemoglobin <=10 mg/dL vs. hemoglobin >10 mg/dL), and how the data are plotted within the application.

*Additionally, the pre-processing steps that must happen before bits of data are merged are not explained and are important. How could a user get a sense of the quality of the data that’s uploaded onto the platform. Provision of the processing steps before data were uploaded would be helpful.*

 Thank you for raising this point. We have added information about the types of checks we do to the manuscript. Briefly, we make sure that IDs match across files, drop variables with no data, identify variables with unexpected values (i.e. character values for a numeric variable), and identify free-text variables that might benefit from standardization and/or translation. We also identify data conflicts when merging files and seek input from the study team as needed.

*The shifting of dates for a given participant is important for data security and privacy. Could the authors discuss the likely impact of this on functions like how can this affect maybe the fitting of longitudinal/time series models?*

 Thank you for the suggestion. We have updated the manuscript to include how shifting dates by up to 7 days forward or backwards introduces noise into longitudinal analysis. Events happening to different people on the same day will appear to have occurred 0-2 weeks apart from each other. However, since the shift is only up to 2 weeks, dates will still cluster within epidemiologically relevant timeframes for most analyses. For analyses requiring exact dates, we recommend users request the original data files.

*Could the authors clarify and broaden the range of study designs that can or cannot be utilized in ClinEpiDB.*

 We have updated the manuscript to highlight the fact that we can support observational study designs (surveillance, cross-sectional, longitudinal cohort, and case-control) as well as randomized control trials. Complex studies that combine elements from different types of study designs require additional query development. These queries may not be able to support all types of exploratory analysis a user would wish to do online, but the website will still support basic data visualization, and users can download the data to do more complex analyses.

*One of the advantages I have observed in ClinEpiDB is the ability to allow users to integrate and visualize different studies. This integration is important to allow analysis of data from multiple sites. However, although the authors promote the use of an ontology-based approach {Open Biological and Biomedical Ontologies}, they do not outline the principles underlying it. Explaining how to deal with integrating distinct studies, mention the ontology-based approach {Open Biological and Biomedical Ontologies} but lack in brief/detail how this ontology works. Does it use a single, hybrid or multiple ontology for instance.*

 We have expanded the description of the ontology-based approach to provide a pointer to the OBO Foundry principles and briefly describe the VEuPathDB ontology, which is an application ontology to hold the OBO Foundry terms that ClinEpiDB uses as well as study-specific terms not available in reference OBO Foundry ontologies.

*The authors include a visualization add on using in a point in click software package which that is very friendly to use. During the use of the software and using Figure 4 as an example, a download button is not available for the graphs produced. Additionally, for one the images produced with plotly on the shiny visuals, only the default download option is available. Maybe perhaps a high res image download option or a reproducible code would be useful to allow one to export high quality graphs for publications or presentations.*

 Thank you for raising this question. We have considered this thoughtfully throughout the development of our website and tools. While our goal is to support data exploration and make research easier, we recognize that drawing conclusions from epidemiology data requires consideration of potential sources of bias and fine-tuned customization depending upon characteristics of each individual dataset. While our visualization tools aim to be as generalizable as possible; designing a fully customizable graph or figure presents challenges. We therefore hope to encourage people to make their own high-quality graphs for presentations and publications during a more thorough, off-line analysis and to use the online tools primarily for initial data exploration purposes. 

*The help page would be more useful as a community forum such that a body of users builds up that can be monitored by the administrator. In this way, users could learn from the queries and responses from other users/administrators and this could save a lot of time for example when similar issues are raised repeatedly.*

 Thank you for the suggestion. We are currently experimenting with adding a tool to our websites that would allow for a community chat where both members of our team and community members could respond to questions as they arise.

*The authors should consider making it a requirement that any publications arising out of data that is generated using this platform is fully open access. This should apply not only to the data, but to the code used to subset and generate the data to allow full transparency and the ability of other users to reproduce analyses and figures.*

 While it is beyond our ability to enforce that publications based on data from ClinEpiDB be open access, we are pleased with the general movement within the community to publish in open access journals, and we agree that code should also be made publicly available on publication. We have added both suggestions to our Data Access and Use Policy. We also added the suggestion that when publishing, users create saved strategies for the data they are publishing on and include the link in the publication, making it easier for others to identify the exact same subset of data.
